# Sex differences in long QT syndrome

**DOI:** 10.3389/fcvm.2023.1164028

**Published:** 2023-04-04

**Authors:** Nuria Díez-Escuté, Elena Arbelo, Estefanía Martínez-Barrios, Patricia Cerralbo, Sergi Cesar, José Cruzalegui, Freddy Chipa, Victoria Fiol, Irene Zschaeck, Clara Hernández, Oscar Campuzano, Georgia Sarquella-Brugada

**Affiliations:** ^1^ Arrhythmia, Inherited Cardiac Diseases and Sudden Death Unit, Hospital Sant Joan de Déu, Barcelona, Spain; ^2^ Arrhythmia Section, Cardiology Department, Hospital Clínic, Universitat de Barcelona, Barcelona, Spain; ^3^ IDIBAPS, Institut d’Investigació August Pi I Sunyer (IDIBAPS), Barcelona, Spain; ^4^ Centro de Investigación Biomédica en Red en Enfermedades Cardiovasculares, Madrid, Spain; ^5^ European Reference Network for Rare, Low Prevalence and Complex Diseases of the Heart, Amsterdam, Netherlands; ^6^ Medical Science Department, School of Medicine, University of Girona, Girona, Spain; ^7^ Cardiovascular Genetics Center, University of Girona-IDIBGI, Girona, Spain

**Keywords:** long QT syndrome, gender, arrhythmias, sudden cardiac death, woman

## Abstract

Long QT Syndrome (LQTS) is a rare, inherited channelopathy characterized by cardiac repolarization dysfunction, leading to a prolonged rate-corrected QT interval in patients who are at risk for malignant ventricular tachyarrhythmias, syncope, and even sudden cardiac death. A complex genetic origin, variable expressivity as well as incomplete penetrance make the diagnosis a clinical challenge. In the last 10 years, there has been a continuous improvement in diagnostic and personalized treatment options. Therefore, several factors such as sex, age diagnosis, QTc interval, and genetic background may contribute to risk stratification of patients, but it still currently remains as a main challenge in LQTS. It is widely accepted that sex is a risk factor itself for some arrhythmias. Female sex has been suggested as a risk factor in the development of malignant arrhythmias associated with LQTS. The existing differences between the sexes are only manifested after puberty, being the hormones the main inducers of arrhythmias. Despite the increased risk in females, no more than 10% of the available publications on LQTS include sex-related data concerning the risk of malignant arrhythmias in females. Therein, the relevance of our review data update concerning women and LQTS.

## 1. Introduction

**Figure 1:**
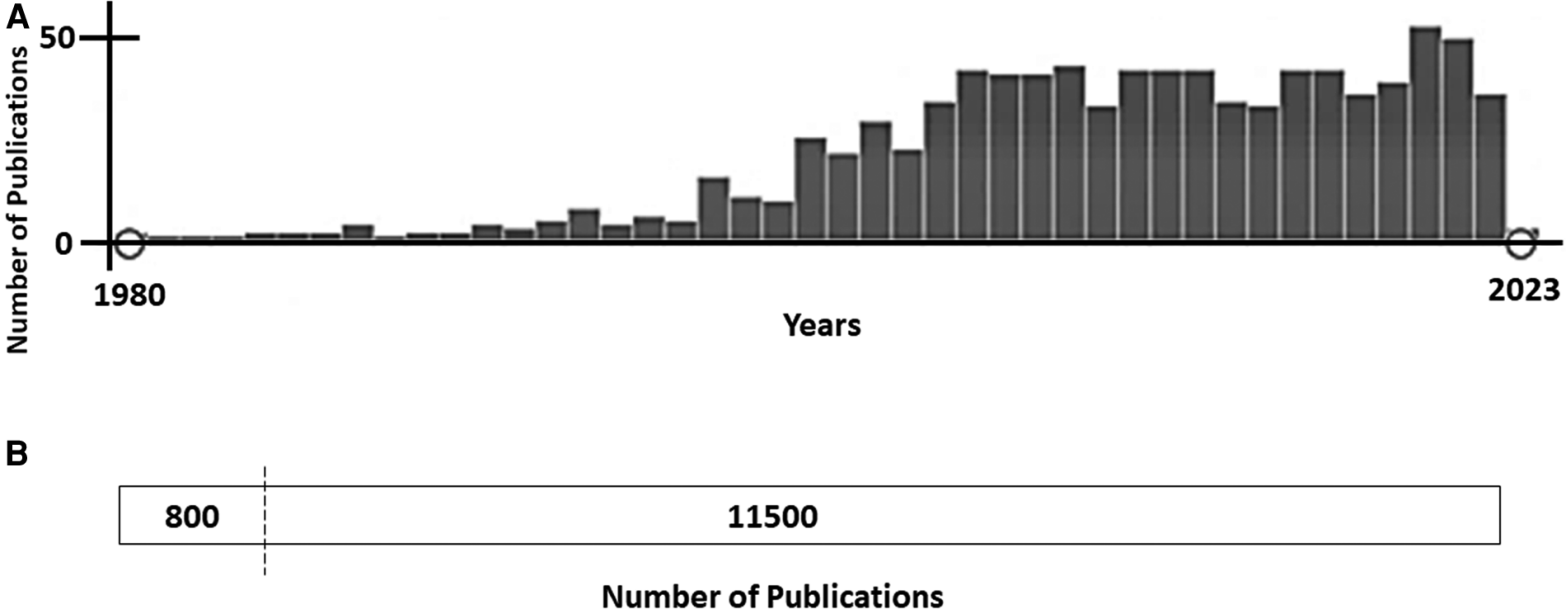
(**A**)- publications focused on long QT syndrome (pubMed, January 2023). From approximately 11,500 publications about Long QT Syndrome, less than 10% (800 publications) included any data concerning females/women or gender/sex differences. (**B**)- Time-line of publications focused on Long QT Syndrome and sex differences (PubMed, January 2023). Since 2005, the number of publications including any data concerning gender/sex differences has been maintained at nearly 45–50 studies per year.

Congenital long QT syndrome (LQTS) is a cardiac channelopathy, characterized by ventricular repolarization and polymorphic ventricular tachycardia (*torsades de pointes*, TdP), leading to malignant arrhythmias, syncope and sudden cardiac death (SCD) at a young age. It is clinically recognized by a prolonged QT interval in the surface electrocardiogram (ECG). LQTS is one of the most common inherited arrhythmia conditions (1:2000/1:2500). LQTS is caused by rare genetic alterations in cardiac ion channels or accessory ion channel subunits, mainly following an autosomal-dominant pattern of inheritance. To date, there are 17 genes potentially associated with LQTS, but definite deleterious alterations have been identified in three genes (*KCNQ1, KCNH2* and *SCN5A*) that account for about 90% of all LQTS cases (1). Due to the potential risk of malignant arrhythmias, it is crucial to accurately identify and manage patients. Continuous advances in diagnosis and personalized treatment, as well as prevention, have been achieved in the last few years. However, risk stratification remains the main, challenge in clinical practice at present. Variable expressivity and incomplete penetrance are hallmarks of LQTS, impeding a conclusive risk stratification. Currently, the existence of differences in sex in LQTS is widely accepted, showing females with an increased risk of developing polymorphic ventricular arrhythmia or SCD than men after the onset of adolescence (2). In addition, women experience a decreased risk during pregnancy, but increased in postpartum period and perimenopause. These differences exist due to hormone levels, which vary depending on the menstrual cycle, gestation, and the postnatal period. Despite being widely accepted as definite risk factor, no more than 10% of studies focused on the pathophysiological mechanism occurring in females suffering from LQTS have been published to date (Figure 1). Herein, we will review evidence from basic and clinical studies involving female-susceptibility to LQTS. A better understanding of the role of sex-related differences in LQTS will lead to improvement in risk stratification.

## 2. Clinical findings

Congenital LQTS is a primary cardiac electric disease characterized by the prolongation of the QT interval, usually associated with T-wave abnormalities. To make a definite diagnosis, it is essential to exclude secondary causes of the prolonged QT interval, such as QT-prolonging drugs or electrolyte imbalances (3). High risk of life-threatening arrhythmias includes T wave alternans and functional 2:1 atrioventricular block in the ECG. In addition to the baseline ECG, QTc behavior can also be assessed during stress testing and 24 h Holter recording, preferably 12-lead. Therefore, the “*Schwartz score*” was developed as a diagnostic criterion to support the diagnosis of the disease if a score of >=3,5 is attained. Adrenaline testing, supine-to-standing ECG or mental stress tests are of lower diagnosis yield (4). It is widely accepted that QT intervals are generally longer in healthy women; therefore, sex-specific cut-off values for prolonged QTc should be applied in order to accurately diagnose LQTS −470 ms in men and 480 ms in women- (5). In addition, women are more susceptible of developing a QT prolongation at slower heart rates, making QTc duration at rest and during sleep critical markers of arrhythmic risk (6). Therefore, adult women with LQTS are at higher risk of malignant arrhythmias due to the influence of a hormone, requiring pharmacological therapy (7). Taking into account all these points, a novel risk score for patients with long QT syndrome has been developed, offering accurate prognostic information to guide clinicians in identifying the patients at the highest risk of life-threatening arrhythmias (8).

The use of β-blockers (preferably nadolol) is the most effective therapy in both sexes (especially in LQTS type 1 and 2), despite the fact that response varies by sex and underlying genotype. Patients with LQTS type3 may benefit from mexiletine or even flecainide; left cardiac sympathetic denervation may be also offered despite rarely and only for special cases. Finally, a reduced number of LQTS patients are suitable for ICD, such as primarily survivors of cardiac arrest and patients at a high-risk for SCD, with recurrent syncope despite adequate pharmacological therapy.

## 3. Genetic and cellular basis

**Figure 2:**
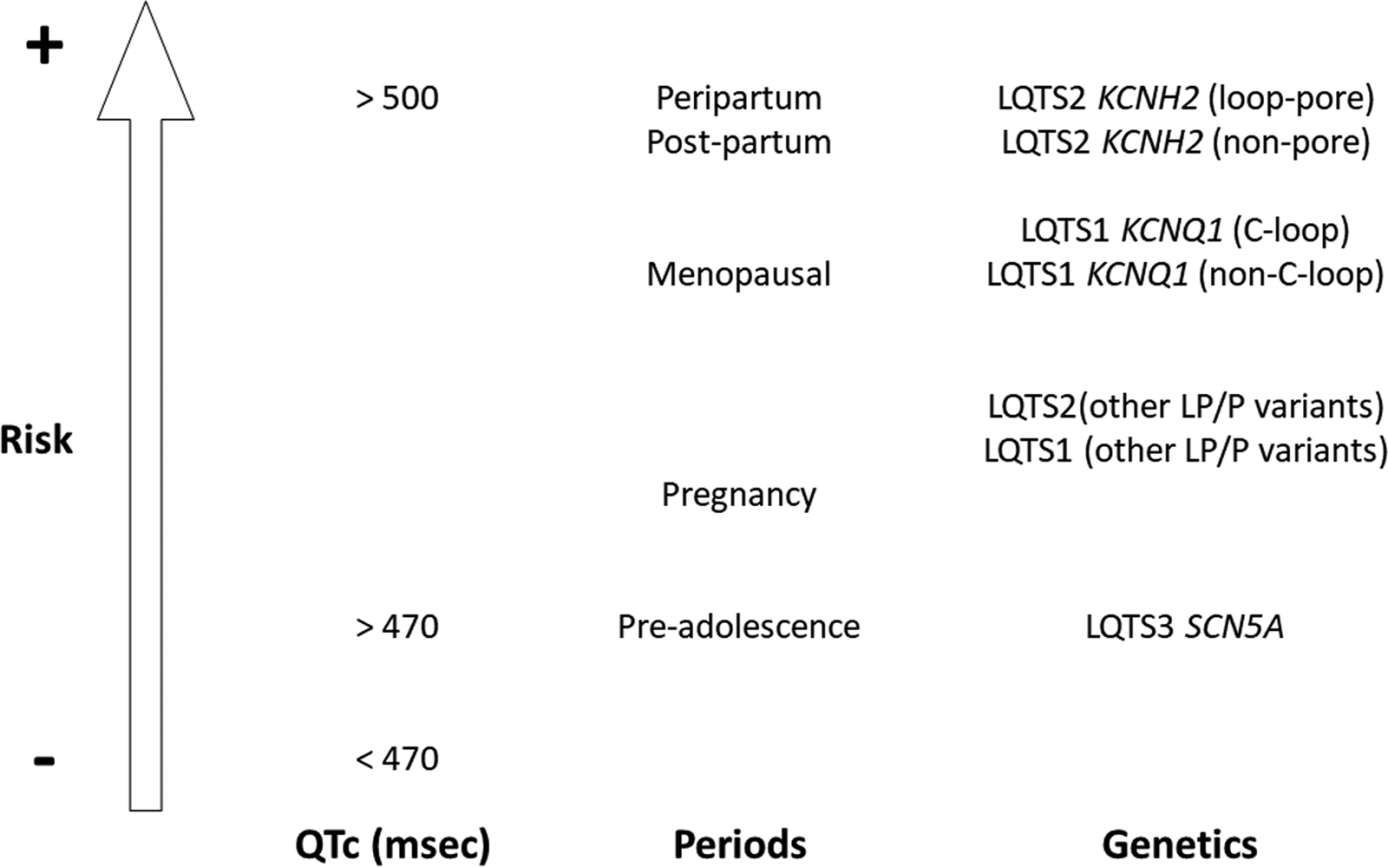
Risk of malignant arrhythmias in women after the onset of adolescence. High risk situations are QTc more than 500msec, peripartum and post-partum periods, and deleterious variants in loop-pore of the *KCNH2* gene. QTc: QT corrected; LQTS: Long QT Syndrome; LP/P: Likely Pathogenic/Pathogenic.

The normal QT interval in the ECG represents the time from the beginning to the end of ventricular depolarization. LQTS is characterized by a prolonged QT interval due to cardiac repolarization dysfunction leading to risk for ventricular tachyarrhythmias, syncope, and even SCD (3). Indeed, QT prolongation is related to a combination of modifiable and unmodifiable risk factors. Electrolyte anomalies are the most common risk factors associated with prolonged QT. Among those, hypokalemia has the main arrhythmogenic effect, as well as hypocalcemia and hypomagnesemia. They may be responsible for prolonging the QT interval, but may also be a major risk factor for drug-induced LQTS, one of the most frequent reasons for QT prolongation (9). Genetic background, as well as older age and female sex, are the most important unmodifiable risk factors. Focused on genetics, rare deleterious alterations located in genes encoding ion channels or associated proteins have been reported as a cause of LQTS. Diagnosed families follow an autosomal dominant pattern of inheritance, with characteristic incomplete penetrance and variable expressivity. Nowadays hundreds of rare alterations have been reported in more than 15 genes (10), despite only a limited number of variants that are definitively classified as deleterious following current ACMG recommendations (11). These causative variants are located mainly in three genes (*KCNQ1, KCNH2,* and *SCN5A*), and are responsible up to 75% of all patients with LQTS (3, 10). Loss-of-function deleterious variants in the potassium channels *KCNQ1* and *KCNH2* are responsible for LQT1 and LQTS2, respectively. They cause decreasing activity of the slow delayed rectifier current (IKs) and rapid delayed rectifier current (IKr) (phase 3 of the action potential), respectively. In contrast, gain-of-function deleterious variants in the *SCN5A* gene (sodium channel, phase 0 of an action potential) are responsible for LQTS3. They cause persistent sodium influx that extends through the plateau phase. A loss of IKs or IKr function, or gain of INa function predisposes ventricular myocytes to early afterdepolarizations, then triggering malignant arrhythmias. Other rare alterations have been reported in minor genes, accounting for 5% of LQTS (*CACNA1C, CALM1, CALM2, CALM3,* and *TRDN*), whereas about 20% of all diagnosed patients do not have an identifiable deleterious variant in any of the current known genes related to LQTS (3). We must also remark that co-inheritance of a second rare variant that affects ventricular repolarization is described as a “second hit”. These variants are classified as deleterious in the same gene (compound heterozygosity) or in a different gene (digenic heterozygosity). Compound deleterious variants are present in 5%–10% of LQTS patients and it is well-accepted as cause of a more severe phenotype (12). Concerning genetic risk after the onset of adolescence, women with pathogenic variants in *KCNQ1* (LQTS1) and *KCNH2* (LQTS2) are at increased risk of malignant arrhythmias, especially LQT2 due to a pore loop pathogenic variant (13) (Figure 2).

## 4. Pregnancy, post-partum and perimenopausal periods

It is widely accepted that there exists a slight female phenotypic predominance in LQTS, with the female sex being a risk factor for malignant arrhythmias. This increased risk occurs after adolescence due to sex hormone differences, with testosterone being a main cause of QT-interval duration in men (2). Low risk of arrhythmias is widely accepted during pregnancy but this risk increases during postpartum, menopausal, and perimenopausal periods (Figure 2). The complex interaction of sex hormones and cardiac ion currents/action potential is widely accepted but it still remains to be clarified (14). What is clear is that sex differences in the electrical substrate are not the result of a simple change in the expression of a single or even a few ionic currents (7). As a preventive measure, a multidisciplinary approach to women with LQTS ensures comprehensive risk assessment and optimal patient care.

### 4.1. Pregnancy

Fluctuations in sex hormone levels during pregnancy could potentially provoke cardiac events. In addition to sex hormone levels, other internal or external factors may alter cardiac electrophysiology during pregnancy and postpartum (alterations in adrenergic activity, disrupted sleep pattern), that can contribute to changes in arrhythmic risk (14). It is widely reported that pregnancy decreases risk of malignant arrhythmias in females diagnosed with LQTS, especially in LQTS type 1 (15). The choice of b-blockers has not been established due to the limited evidence available nowadays. One thing that is for certain is that the use of b-blockers such as propranolol is effective in reducing the risk of arrhythmic events (15, 16). However, lower fetal birth weight has been reported, leading to non-selective b-blocker use, mainly metoprolol (17). Because of the risk in mothers with LQTS for stillbirth and the higher risk of miscarriages, a more stringent follow-up of these patients during pregnancy might be necessary. Finally, concerning contraceptives, no conclusive studies have been published to date, with the risk of arrhythmias remaining unknown (18).

### 4.2. Postpartum

Concerning post-partum follow-up, women with LQTS, especially LQTS type 2, show a higher risk of VT and SCD in comparison to the relatively low risk during pregnancy. After postpartum, the risk of cardiac events returns to basal levels before pregnancy. However, there are no general recommendations and approved schemes on how women with LQTS should be supervised after delivery. Limited data suggest increased risk in the early post-partum period, particularly in patients with LQTS2 (15). In addition, the use of b-blockers is mostly well tolerated during the postpartum period to prevent life-threatening manifestations. It is also important to note that b-blockers are secreted in breast milk, but hypoglycemia and bradycardia may occur in breastfed infants, albeit rarely (19). Therefore, dose adjustment of beta-blockers may be needed and postpartum care remains the same as in routine cases (20).

### 4.3. Menopausal and perimenopausal periods

Nowadays, only one study has been published focusing on females after the onset of menopause who have been diagnosed with LQTS. Available data suggest a higher risk of malignant arrhythmias in LQTS-type2 women (21). In contrast, there are studies in post-menopausal period showing that estrogen replacement therapy (ERT) prolongs the QTc interval more, compared to those postmenopausal women taking no hormones or taking combined estrogen-progestin replacement therapies (22, 23). It suggests that progesterone has a similar protective effect to testosterone (24).

## 5. Role of hormones in arrhythmogenic risk

The underlying molecular mechanisms that cause patients with LQTS to have sex-dependent variability in arrhythmogenic risk remain to be elucidated. However, several studies suggest that it results from the effect of certain hormones on cardiac ion channels. Animal experiments have shown that estradiol could act as a pro-arrhythmic agent in LQTS2, whereas progesterone would have a protective role (25). This effect of estradiol is attributed to its interaction with certain potassium ion currents (26), and due to the effect on the transcription of some genes like *KCNE2* and *RyR2*, being mediated by an estrogen receptor-dependent process (27, 28). However, such effects have not been supported in human studies, in which a more complex interaction between estrogen and progesterone appears to exist (29). For example, a recent study in women with LQTS found an inverse relationship of RR interval with estradiol levels during the menstrual cycle (30). In addition, progesterone was found to show an inverse association with the corrected QT interval and with the ratio of progesterone to estradiol in women with LQTS2. This shortening of the QT interval was observed during the luteal phase, and was mainly attributed to increased progesterone levels in this phase (30). Such associations were not maintained in women with LQTS1, supporting that the observed differences in LQTS subtypes are due to different effects of hormones on the channels, with different sensitivity between genotypes. Likewise, it has been shown that progesterone may have anti-arrhythmic effects, which may help to reduce the risk of arrhythmias (31). These effects of progesterone on the QT interval are related to an increase in intracellular calcium reuptake from the sarcoplasmic reticulum mediated by SERCA2a (32). Similar to progesterone, testosterone decreases I_CaL_ current and increases potassium channel currents (I_Kr_, I_Ks_, I_K1_), reducing the QTc interval in animals and humans (33–35).

During pregnancy, the combination of hormones is complex. Apart from the interaction of estrogen and progesterone, oxytocin released towards the end of pregnancy, during labor and lactation, would have direct effects on sodium channels and cause a direct acute inhibition on the potassium I_Ks_ channel (36), which could explain the increased risk in post-partum women with LQTS2. Data on the potential impact of prolactin on cardiac electrophysiology are limited, but some animal studies suggest that it would act similarly to oxytocin (36).

## 6. Conclusions

LQTS is a rare heterogenous group of arrhythmogenic entities, characterized by a prolonged QT interval. In recent years, a continuous improvement in diagnosis as well as genetic/pathophysiological mechanism has been performed, however, risk stratification remains a current challenge. It is widely accepted that sex is an independent risk factor due to females having a high risk of malignant arrhythmias associated with LQTS. Hormones seem to be the main reason for reported sex differences, despite the fact that the link between the sex hormones and susceptibility to malignant arrhythmias is still a matter of debate. Additional studies will help to unravel the pathophysiological mechanism involved in sex differences, helping to define a proper risk stratification in LQTS patients.

## References

[1] Wilde AAM Amin AS Postema PG. Diagnosis, management and therapeutic strategies for congenital long QT syndrome. Heart. (2022) 108:332–8. 10.1136/heartjnl-2020-318259 34039680 PMC8862104

[2] Costa S Saguner AM Gasperetti A Akdis D Brunckhorst C Duru F. The link between sex hormones and susceptibility to cardiac arrhythmias: from molecular basis to clinical implications. Front Cardiovasc Med. (2021) 8:644279. 10.3389/fcvm.2021.644279 33681311 PMC7925388

[3] Wilde AAM Amin AS. Clinical Spectrum of SCN5A mutations: long QT syndrome. Brugada Syndrome, and Cardiomyopathy. JACC Clin Electrophysiol. (2018) 4:569–79. 10.1016/j.jacep.2018.03.006 29798782

[4] Lankaputhra M Voskoboinik A. Congenital long QT syndrome: a clinician's Guide. Intern Med J. (2021) 51:1999–2011. 10.1111/imj.15437 34151491

[5] Vink AS Neumann B Lieve KVV Sinner MF Hofman N El Kadi S Determination and interpretation of the QT interval. Circulation. (2018) 138:2345–58. 10.1161/CIRCULATIONAHA.118.033943 30571576

[6] Wilde AAM Semsarian C Marquez MF Sepehri Shamloo A Ackerman MJ Ashley EA European Heart rhythm association (EHRA)/heart rhythm society (HRS)/Asia pacific heart rhythm society (APHRS)/latin American heart rhythm society (LAHRS) expert consensus statement on the state of genetic testing for cardiac diseases. Heart Rhythm. (2022) 19(7):e1–60. 10.1016/j.hrthm.2022.03.1225 35390533

[7] Salama G Bett GC. Sex differences in the mechanisms underlying long QT syndrome. Am J Physiol Heart Circ Physiol. (2014) 307:H640–8. 10.1152/ajpheart.00864.2013 24973386 PMC4187395

[8] Mazzanti A Trancuccio A Kukavica D Pagan E Wang M Mohsin M Independent validation and clinical implications of the risk prediction model for long QT syndrome (1-2-3-LQTS-risk). Europace. (2022) 24:614–9. 10.1093/europace/euab238 34505884

[9] Li Z Guo X Guo L Zheng L Yu S Yang H Sex differences in association between decreased glomerular filtration rate and prolongation of corrected QT interval in general Chinese population. Eur J Intern Med. (2017) 43:e33–5. 10.1016/j.ejim.2017.05.011 28511851

[10] Adler A Novelli V Amin AS Abiusi E Care M Nannenberg EA An international, multicentered, evidence-based reappraisal of genes reported to cause congenital long QT syndrome. Circulation. (2020) 141:418–28. 10.1161/CIRCULATIONAHA.119.043132 31983240 PMC7017940

[11] Richards S Aziz N Bale S Bick D Das S Gastier-Foster J Standards and guidelines for the interpretation of sequence variants: a joint consensus recommendation of the American college of medical genetics and genomics and the association for molecular pathology. Genet Med. (2015) 17:405–24. 10.1038/gim.2015.30 25741868 PMC4544753

[12] Etheridge SP Asaki SY Niu MC. A personalized approach to long QT syndrome. Curr Opin Cardiol. (2019) 34:46–56. 10.1097/HCO.0000000000000587 30394905

[13] Goldenberg I Bos JM Yoruk A Chen AY Lopes C Huang DT Risk prediction in women with congenital long QT syndrome. J Am Heart Assoc. (2021) 10:e021088. 10.1161/JAHA.121.021088 34238014 PMC8483453

[14] Asatryan B Yee L Ben-Haim Y Dobner S Servatius H Roten L Sex-Related differences in cardiac channelopathies: implications for clinical practice. Circulation. (2021) 143:739–52. 10.1161/CIRCULATIONAHA.120.048250 33587657

[15] Seth R Moss AJ McNitt S Zareba W Andrews ML Qi M Long QT syndrome and pregnancy. J Am Coll Cardiol. (2007) 49:1092–8. 10.1016/j.jacc.2006.09.054 17349890

[16] Ishibashi K Aiba T Kamiya C Miyazaki A Sakaguchi H Wada M Arrhythmia risk and beta-blocker therapy in pregnant women with long QT syndrome. Heart. (2017) 103:1374–9. 10.1136/heartjnl-2016-310617 28292826

[17] Regitz-Zagrosek V Roos-Hesselink JW Bauersachs J Blomstrom-Lundqvist C Cifkova R De Bonis M 2018 ESC guidelines for the management of cardiovascular diseases during pregnancy. Eur Heart J. (2018) 39:3165–241. 10.1093/eurheartj/ehy340 30165544

[18] Goldenberg I Younis A Huang DT Yoruk A Rosero SZ Cutter K Use of oral contraceptives in women with congenital long QT syndrome. Heart Rhythm. (2022) 19:41–8. 10.1016/j.hrthm.2021.07.058 34339849

[19] de Bruin R van Dalen SL Franx SJ Simons SH Flint RB van den Bosch GE. Risk for neonatal hypoglycaemia and bradycardia after beta-blocker use during pregnancy or lactation: a systematic review and meta-analysis protocol. BMJ open. (2022) 12:e055292. 10.1136/bmjopen-2021-055292 36008071 PMC9422831

[20] Marcinkeviciene A Rinkuniene D Puodziukynas A. Long QT syndrome management during and after pregnancy. Medicina (Kaunas). (2022) 58(11):1694. 10.3390/medicina58111694 36422233 PMC9696301

[21] Buber J Mathew J Moss AJ Hall WJ Barsheshet A McNitt S Risk of recurrent cardiac events after onset of menopause in women with congenital long-QT syndrome types 1 and 2. Circulation. (2011) 123:2784–91. 10.1161/CIRCULATIONAHA.110.000620 21632495 PMC3155756

[22] Haseroth K Seyffart K Wehling M Christ M. Effects of progestin-estrogen replacement therapy on QT-dispersion in postmenopausal women. Int J Cardiol. (2000) 75:161–5; discussion 165–6. 10.1016/S0167-5273(00)00317-X 11077128

[23] Kadish AH Greenland P Limacher MC Frishman WH Daugherty SA Schwartz JB. Estrogen and progestin use and the QT interval in postmenopausal women. Ann Noninvasive Electrocardiol. (2004) 9:366–74. 10.1111/j.1542-474X.2004.94580.x 15485516 PMC6932472

[24] Schwartz JB Volterrani M Caminiti G Marazzi G Fini M Rosano GM Effects of testosterone on the Q-T interval in older men and older women with chronic heart failure. Int J Androl. (2011) 34:e415–21. 10.1111/j.1365-2605.2011.01163.x 21615419

[25] Odening KE Choi BR Liu GX Hartmann K Ziv O Chaves L Estradiol promotes sudden cardiac death in transgenic long QT type 2 rabbits while progesterone is protective. Heart Rhythm. (2012) 9:823–32. 10.1016/j.hrthm.2012.01.009 22245795 PMC4397932

[26] Kurokawa J Kodama M Furukawa T Clancy CE. Sex and gender aspects in antiarrhythmic therapy. Handb Exp Pharmacol. (2012):237–63. 10.3389/fcvm.2021.644279 23027454

[27] Kundu P Ciobotaru A Foroughi S Toro L Stefani E Eghbali M. Hormonal regulation of cardiac KCNE2 gene expression. Mol Cell Endocrinol. (2008) 292:50–62. 10.1016/j.mce.2008.06.003 18611433 PMC2893227

[28] Long V Fiset C. Contribution of estrogen to the pregnancy-induced increase in cardiac automaticity. J Mol Cell Cardiol. (2020) 147:27–34. 10.1016/j.yjmcc.2020.08.005 32798536

[29] Vink AS Clur SB Wilde AAM Blom NA. Effect of age and gender on the QTc-interval in healthy individuals and patients with long-QT syndrome. Trends Cardiovasc Med. (2018) 28:64–75. 10.1016/j.tcm.2017.07.012 28869094

[30] Bjelic M Zareba W Peterson DR Younis A Aktas MK Huang DT Sex hormones and repolarization dynamics during the menstrual cycle in women with congenital long QT syndrome. Heart Rhythm. (2022) 19:1532–40. 10.1016/j.hrthm.2022.04.029 35525425

[31] Asatryan B Rieder M Castiglione A Odening KE. Arrhythmic risk during pregnancy and postpartum in patients with long QT syndrome. Herzschrittmacherther Elektrophysiol. (2021) 32:180–5. 10.1007/s00399-021-00757-4 33782754 PMC8166676

[32] Moshal KS Zhang Z Roder K Kim TY Cooper L Patedakis Litvinov B Progesterone modulates SERCA2a expression and function in rabbit cardiomyocytes. Am J Physiol Cell Physiol. (2014) 307:C1050–7. 10.1152/ajpcell.00127.2014 25252951 PMC4254949

[33] Montano LM Calixto E Figueroa A Flores-Soto E Carbajal V Perusquia M. Relaxation of androgens on rat thoracic aorta: testosterone concentration dependent agonist/antagonist L-type Ca2 + channel activity, and 5beta-dihydrotestosterone restricted to L-type Ca2 + channel blockade. Endocrinology. (2008) 149:2517–26. 10.1210/en.2007-1288 18276759

[34] Masuda K Takanari H Morishima M Ma F Wang Y Takahashi N Testosterone-mediated upregulation of delayed rectifier potassium channel in cardiomyocytes causes abbreviation of QT intervals in rats. J Physiol Sci. (2018) 68:759–67. 10.1007/s12576-017-0590-4 29332211 PMC10717990

[35] Gutierrez G Wamboldt R Baranchuk A. The impact of testosterone on the QT interval: a systematic review. Curr Probl Cardiol. (2022) 47:100882. 10.1016/j.cpcardiol.2021.100882 34103195

[36] Bodi I Sorge J Castiglione A Glatz SM Wuelfers EM Franke G Postpartum hormones oxytocin and prolactin cause pro-arrhythmic prolongation of cardiac repolarization in long QT syndrome type 2. Europace. (2019) 21:1126–38. 10.1093/europace/euz037 30938413

